# Human induced pluripotent stem cells derived neutrophils display strong anti-microbial potencies

**DOI:** 10.1186/s13619-025-00227-z

**Published:** 2025-03-21

**Authors:** Xing Hu, Baoqiang Kang, Mingquan Wang, Huaisong Lin, Zhiyong Liu, Zhishuai Zhang, Jiaming Gu, Yuchan Mai, Xinrui Guo, Wanli Ma, Han Yan, Shuoting Wang, Jingxi Huang, Junwei Wang, Jian Zhang, Tianyu Zhang, Bo Feng, Yanling Zhu, Guangjin Pan

**Affiliations:** 1 National Key Laboratory of Immune Response and Immunotherapy, Guangzhou Institutes of Biomedicine and Health, Chinese Academy of Sciences, Guangzhou, 510530 China; 2 University of Chinese Academy of Sciences, Beijing, 100049 China; 3 Centre for Regenerative Medicine and Health, Hong Kong Institute of Science and Innovation, Chinese Academy of Sciences, Hong Kong, China; 4 Guangdong Provincial Key Laboratory of Stem Cell and Regenerative Medicine, Guangdong-Hong Kong Joint Laboratory for Stem Cell and Regenerative Medicine, Center for Development and Regeneration, Guangzhou Institutes of Biomedicine and Health, Chinese Academy of Sciences, Guangzhou, 510530 China; 5 GIBH-HKU Guangdong-Hong Kong Stem Cell and Regenerative Medicine Research Centre, GIBH-CUHK Joint Research Laboratory on Stem Cell and Regenerative Medicine, Guangzhou Institutes of Biomedicine and Health, Chinese Academy of Sciences, 510530 Guangzhou, China; 6 School of Biomedical Sciences, Faculty of Medicine, CUHK-GIBH CAS Joint Research Laboratory on Stem Cell and Regenerative Medicine, The Chinese University of Hong Kong, Room 105A, Lo Kwee-Seong Integrated Biomedical Sciences Building, Area 39, Shatin, NT, Hong Kong SAR, China; 7 The Third Affiliated Hospital of Sun Yat-Sen University, Guangzhou, 510000 China

**Keywords:** hiPSCs, Neutrophils, Bacteria, Cell therapy

## Abstract

Neutrophils are essential innate immune cells with unusual anti-microbial properties while dysfunctions of neutrophils lead to severe health problems such as lethal infections. Generation of neutrophils from human induced pluripotent stem cells (hiPSCs) is highly promising to produce off-the-shelf neutrophils for transfusion therapies. However, the anti-microbial potencies of hiPSCs derived neutrophils (iNEUs) remain less documented. Here, we develop a scalable approach to generate iNEUs in a chemical defined condition. iNEUs display typical neutrophil characters in terms of phagocytosis, migration, formation of neutrophil extracellular traps (NETs), etc. Importantly, iNEUs display a strong killing potency against various bacteria such as *K.pneumoniae*, *P.aeruginosa, E.coli* and *S.aureus*. Moreover, transfusions of iNEUs in mice with neutrophil dysfunction largely enhance their survival in lethal infection of different bacteria. Together, our data show that hiPSCs derived neutrophils hold strong anti-microbial potencies to protect severe infections under neutrophil dysfunction conditions.

**Supplementary Information:**

The online version contains supplementary material available at 10.1186/s13619-025-00227-z.

## Background

Neutrophils are critical component of the innate immune defense system against microbial infections in human (Liew and Kubes 2019). Neutrophils also play important roles to maintain the immune homeostasis through cross talking with other immune cells, such as macrophages (Marwick et al. 2018; Uderhardt et al. 2019). Prolonged dysfunction of neutrophils such as neutropenia that are usually caused by chemotherapy, radiotherapy or hematologic diseases often leads to life threaten infections despite continued progression of antibacterial therapies (Hopff et al. 2024; Murray et al. 2022; Schwartzberg 2006). Neutrophil dysregulation also leads to other severe health problems such as uncontrolled inflammation, auto-immune diseases as well as cancer (Casanova et al. 2024; Mollinedo 2019; Wright et al. 2010). Neutrophil transfusion is potential to benefit the patients with neutropenia (Estcourt et al. 2015; Seidel et al. 2008), especially for those patients infected with antibiotic resistant bacteria. However, limitations of collecting enough doses of functional neutrophils for transfusion largely hamper the clinical progress of neutrophil therapy.

Human induced pluripotent stem cells (hiPSCs) have the unique unlimited self-renewal and pluripotency to differentiate into other somatic cell types, thus offering a scalable source to generate off-the-shelf neutrophils. Differentiation of hiPSCs for neutrophils (iNEUs) has been attempted by different groups (Brok-Volchanskaya et al. 2019; Chang et al. 2022; Lachmann et al. 2015; Miyauchi et al. 2021; Saeki et al. 2009; Sweeney et al. 2016; Trump et al. 2019; Yokoyama et al. 2009). Slukvin’s group reported a differentiation protocol to generate myeloid and then neutrophils through a hiPSCs derived hemogenic endothelium intermediate by ETV2 mRNA (Brok-Volchanskaya et al. 2019). Another group reported a protocol to generate expandable hiPSCs derived neutrophil-primed progenitors (NeuPs) by over-expression of *C-MYC* and *BMI1* (Miyauchi et al. 2021). Inactivation of these transgenes allowed them to obtain neutrophils that can promote the survival of infected mice through transfusion (Miyauchi et al. 2021). However, the biological functions of the in vitro generated iNEUs, especially their anti-microbial properties remain less documented.

We previously reported a defined and monolayer condition to generate hiPSCs derived hematopoietic stem/progenitor cells (iHSPCs) with multipotency to differentiate into various subtype blood/immune cells (Brok-Volchanskaya et al. 2019; Gu et al. 2024; Zhang et al. 2019). These iHSPCs have preferential myeloid differentiation potency and could be continuously collected as floating cells in the monolayer hiPSCs differentiation culture for more than 10 days (Li et al. 2024; Zhang et al. 2019; Zhu et al. 2020), thus provide an efficient and easy approach to obtain iHSPCs.

Here in this study, we further trigger myeloid differentiation of iHSPCs to generate functional neutrophils. The iNEUs display typical neutrophil characters and a strong anti-microbial potency against various bacteria and promote the survival of mice with neutrophil dysfunction in lethal infection with different bacteria.

## Results

### Generation of neutrophils from hiPSCs

**Fig. 1:**
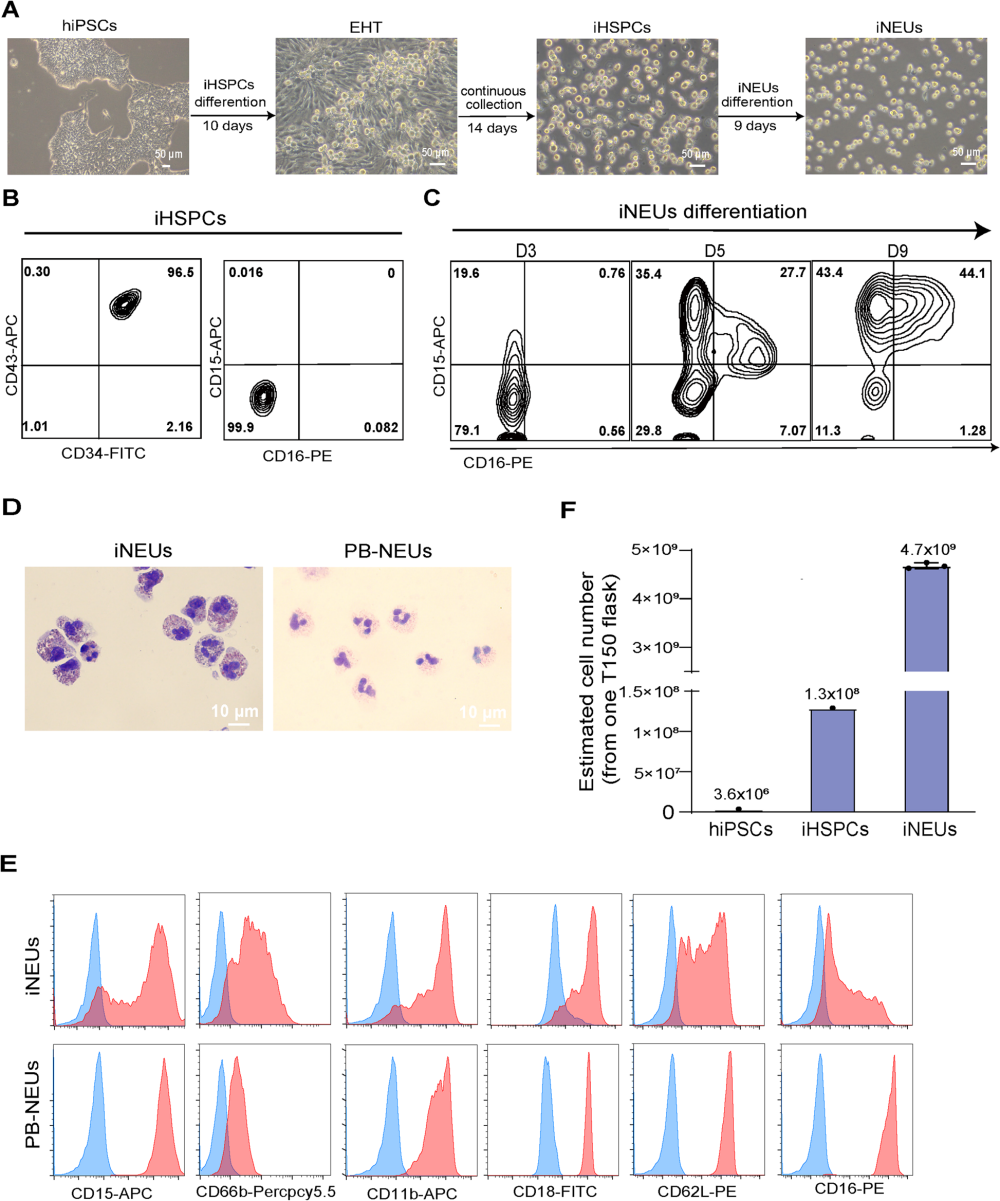
Generation of neutrophils from hiPSCs. **A** Representative brightfield images of different stages of iNEUs differentiation from hiPSCs. Scale bar, 50 μm. **B**, **C** Representative FACS data of indicated markers on iHSPCs (**B**) and iNEUs differentiation at different time points (**C**). **D** Wright–Giemsa images of iNEUs and PB-NEUs. Scale bar, 10 μm. **E** Representative FACS data of indicated surface markers (CD15, CD66b, CD11b, CD18, CD62L and CD16; red filled) on iNEUs and PB-NEUs (blue, respective isotype control). **F** Estimated number of iHSPCs and iNEUs obtained from 3 × 10^6^ hiPSCs cultured in 1 T150 flask

We previously reported a defined, monolayer approach to generate iHSPCs from hiPSCs (Gu et al. 2024; Kang et al. 2024; Kang et al. 2022; Li et al. 2024; Zhang et al. 2019; Zhu et al. 2020). iHSPCs were generated through a typical endothelial-hematopoietic-transition (EHT) process which allows a continued collection of floating iHSPCs in the attached differentiation culture (Fig. 1A). For neutrophil generation, we collected iHSPCs and then cultured them in the presence of myeloid cytokines G-CSF and IL3 (Methods). iHSPCs showed a homogenous population expressing HPSC markers such as CD34^+^/CD43^+^, but negative for myeloid markers such as CD15^−^/CD16^−^ (Fig. 1B). Upon myeloid differentiation, the cells gradually acquired myeloid markers and around 90% of cells were positive for CD15 and/or CD16 at 9 days differentiation (Fig. 1C). iNEUs displayed a typical neutrophil morphology with segmented nuclei, which is comparable to human neutrophils from peripheral blood (PB-NEUs) (Fig. 1D). Other surface markers related to neutrophil functions such as CD66b, CD62L, etc. were also well expressed in iNEUs (Fig. 1E). We further calculated the efficiency of iNEU generation. When starting with 1 T150 flask containing 3 × 10^6^~4 × 10^6^ hiPSCs, we could generate roughly 1 × 10^8^~2 × 10^8^ iHPSCs and then 4 × 10^9^~6 × 10^9^ iNEUs (Fig. 1F). In sum, we develop a defined and scalable approach to generate iNEUs.

### iNEUs have typical neutrophil characters

**Fig. 2:**
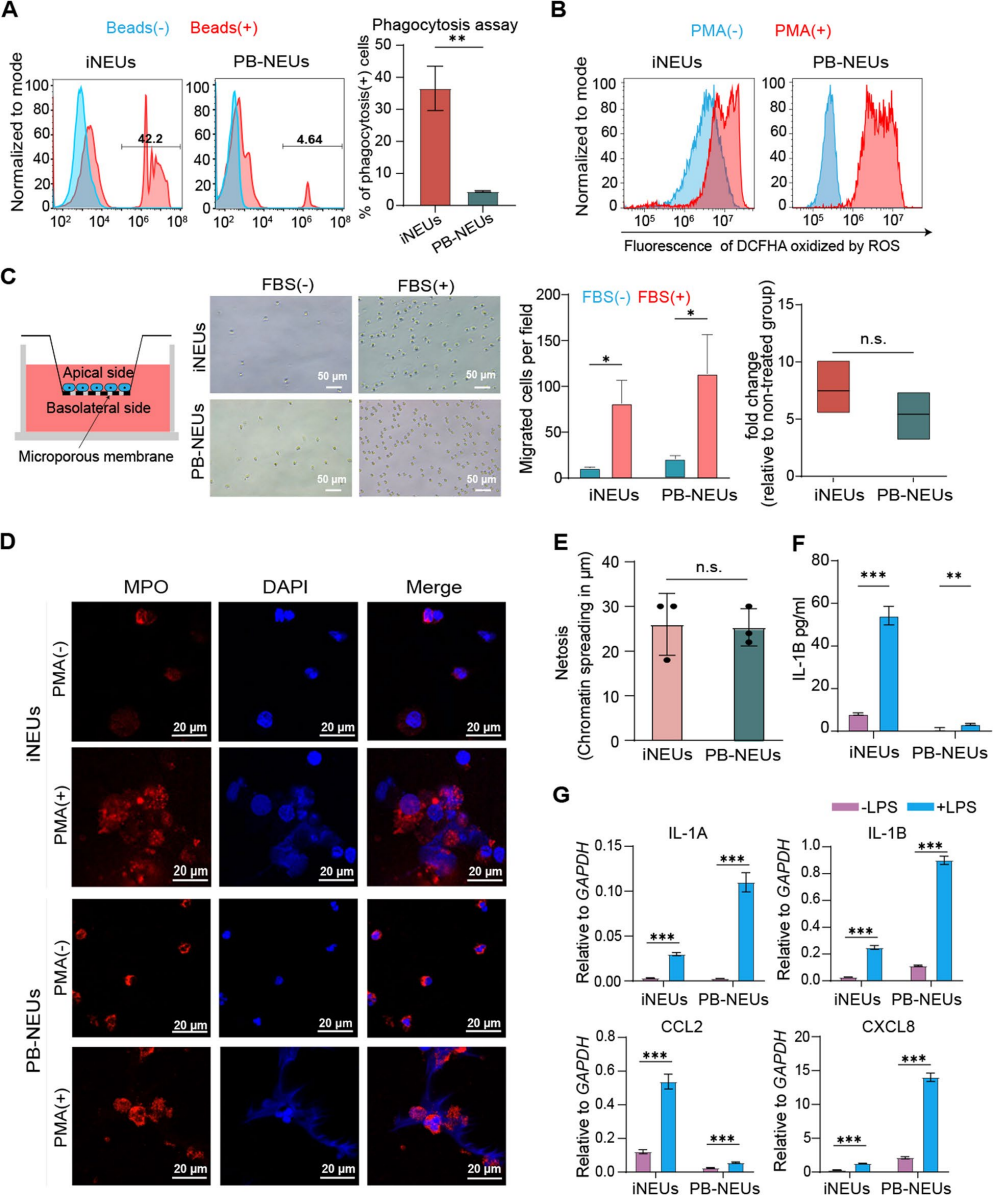
Characterizations of iNEUs. **A** Phagocytosis of red fluorescent-labeled latex beads by neutrophils analyzed by flow cytometry. Left: diagrams of a representative experiment (red filled, cells treated with 1 mm beads; blue, untreated control). Right graph: percentage of phagocytosing cells, *n* = 3 independent experiments; mean ± SD. **P < 0.01. **B** Representative flow cytometry of oxidative burst assay in neutrophils with or without phorbol myristate acetate (PMA) stimulation, through reactive oxygen species (ROS) analysis kit. **C** Migration analysis of neutrophils. Far left: schematic of transwell chamber model. Second panel: bright field image of cells in the lower chamber after 24 h of cell incubation in the transwell chambers. Scale bar, 50 μm. Third panel: cell counts in each bright field image under a random field of view. Right panel: the relative migration in the FBS-primed cell group compared to the no FBS group. *n* = 3 independent experiments; mean ± SD. *P < 0.05, ns, not significant. **D** Confocal fluorescence microscopy of neutrophil extracellular trap (NETs) formation by neutrophils before or after 7-h PMA stimulation. Scale bar, 20 μm. **E** Quantification of chromatin spreading (maximum length) during NET formation. ns, not significant. **F** Protein levels of IL-1b in iNEUs with or without LPS stimulation, using Elisa. *n* = 3 independent experiments. **P < 0.05, ***P < 0.001. **G** Gene expression of chemokines and cytokines in iNEUs with or without LPS stimulation, using qPCR. *n* = 3 independent experiments, normalized to GAPDH expression. ***P < 0.001

We then examined antibacterial functions of iNEUs in terms of phagocytosis, ROS production, migration, as well as formation of NETs. iNEUs showed significant phagocytosis potency to engulf PE labeled beads, which is comparable to or even better than the primary neutrophils isolated from human peripheral blood (PB-NEUs) (Fig. 2A). iNEUs also showed ROS production and oxidative burst upon PMA stimulation, albeit to a less extent than PB-NEUs (Fig. 2B). An important biological function of neutrophils is chemotactic migration. Based on a trans-well assay, iNEUs showed a good migration attracted by fetal bovine serum (FBS), comparable to PB-neutrophils (Fig. 2C). NETs formation is important for the antibacterial functions of neutrophils. We confirmed that iNEUs could form typical NETs upon PMA stimulation (Fig. 2D) and the netosis sizes were comparable to PB-iNEUs (Fig. 2E). Lastly, iNEUs also showed induced expression of important cytokines for neutrophil functions upon stimulation by LPS (Fig. 2F-G). In all, we demonstrate that iNEUs have typical neutrophil characters which are comparable to primary neutrophils from human peripheral blood.

### iNEUs resemble PB-neutrophils rather than macrophages in transcriptome

**Fig. 3:**
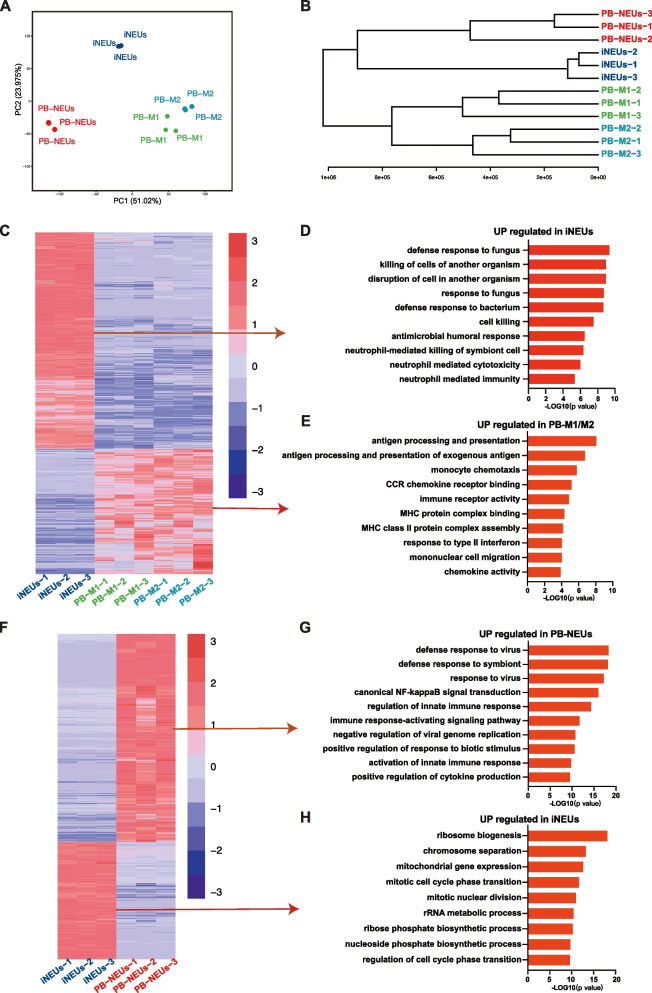
Gene expression profiling of iNEUs. **A** Principal component analysis of the gene expression in iNEUs, PB-NEUs, PB-M and PB-M2. **B** Hierarchical clustering of RNA sequencing (RNA-seq) expression data of iNEUs, PB-NEUs and PB-M1/M2. **C** Heatmap of the differentially expressed genes in indicated cells. **D** Up-regulated genes in iNEUs by gene ontology analysis. **E** Up-regulated genes in PB-M1/M2 by gene ontology analysis. **F** Heatmap of the differentially expressed genes in indicated cells. **G** Up-regulated genes in PB-NEUs by gene ontology analysis. **H** Up-regulated genes in iNEUs by gene ontology analysis

To further analyze iNEUs, we performed RNA-seq analysis on iNEUs as well as PB-neutrophils and M1 or M2 PB-macrophages (Fig. 3A). The transcriptome of iNEUs were clustered together with PB-neutrophils while not M1 or M2 PB-macrophages (Fig. 3A-B). The genes that were up-regulated in iNEUs compared with PB-macrophages were enriched in typical neutrophil functions (Fig. 3C-D). In contrast, the genes up-regulated in PB-macrophages were enriched in adaptive immune functions, such as antigen presentation (Fig. 3E). Compared with PB-neutrophils, the gene up-regulated in iNEUs were enriched in the active biogenesis functions (Fig. 3F), indicating the stem cells differentiated iNEUs hold more proliferation capabilities with the mature PB-neutrophils. While PB-neutrophils showed much more innate immunity functions such as virus defense as well as cytokine production (Fig. 3F-H). In all, we showed that the stem cell derived iNEUs resemble neutrophils from human peripheral blood in transcriptome.

### iNEUs have strong antibacterial potency against different bacteria

**Fig. 4:**
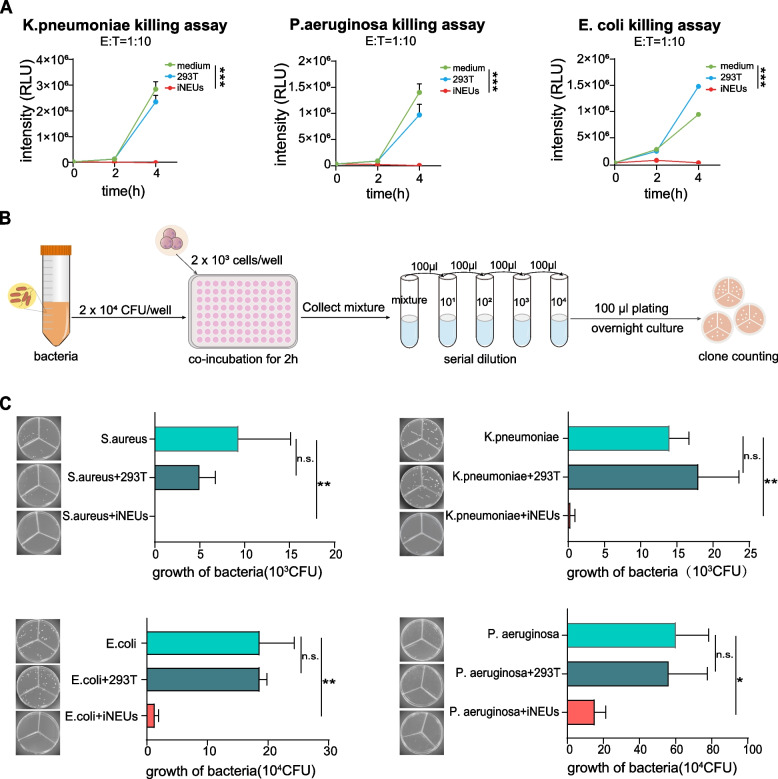
iNEUs exhibit strong antimicrobial potency. **A** RLU counts in indicated bacteria co-culturing with iNEUs at 2 or 4 h. At 0 h, 5 × 10^4^ RLUs of bacteria were mixed with 5 × 10^4^ iNEUs for co-culturing, E: T = 1:10 (1 RLU of bacteria is equivalent to 10 CFUs of bacteria). iNEUs, experimental group; medium, blank control group; 293 T: negative control group. *n* = 5 independent experiments; mean ± SD, ***P < 0.001. **B** Flowchart of the bacterial killing plate assay. **C** Colony forming units (CFUs) formed by indicated bacteria preincubated with indicated cells. Upon 2 h of co-culturing of the bacteria and cells, the mixture was performed a serial dilution for plating and cultured overnight for CFU formation. At 0 h, 2 × 10^4^ CFUs of bacteria were mixed with 2 × 10^3^ cells, E: T = 10. *n* = 3; mean ± SD, *P < 0.05, **P < 0.01, ns, not significant

Whether hiPSCs derived neutrophils have antibacterial potency against different types of bacteria remains less extensively examined. We then sought to analyze the antibacterial functions of iNEUs in the context of different bacteria including gram positive (G+) and negative (G-) ones. Firstly, based on a bioluminescence labeled killing assay (Manganelli et al. 2015; Tyagi et al. 2012), iNEUs displayed a strong suppressive ability on *K. pneumoniae, P. aeruginosa* as well as *E. coli* (Fig. 4A). To examine the bacteria killing more directly, iNEUs were incubated with different bacteria in an effector-to-target (E: T) ratio of 1:10 for 2 h and then plated for the CFUs formation assay (Fig. 4B). As shown in Fig. 4C, bacteria which were pre-incubated with iNEUs for 2 h showed much reduced or even no CFU formation (Fig. 4C). These data demonstrate that iNEUs display a strong antibacterial potency against different bacteria including G+ and G- bacteria.

### iNEUs transfusion protects the mice with impaired neutrophil function under lethal infections

**Fig. 5:**
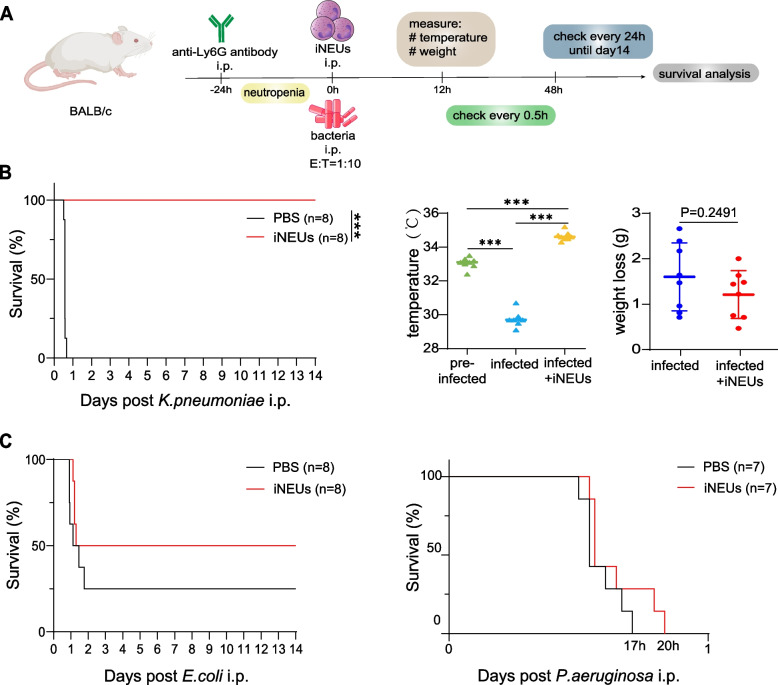
iNEUs transfusion promotes survival of mice with impaired neutrophil under lethal infections. **A** Schematic of the experiment design. The mice were treated by anti-Ly6G antibody to impair neutrophils and were intraperitoneally injected with bacteria with or without iNEUs. Bacteria Dose: 5 × 10^8^ CFUs *E. coli*, 5 × 10^7^ CFUs *P. aeruginosa,* or 5 × 10^7^ CFUs *K. pneumoniae*. The number of cells was one tenth of bacteria, E: T = 1:10. **B** Survival rates, body temperature and weight loss at indicated groups of mice. Left, Kaplan–Meier survival curve of neutrophil-depleted BALB/c mice with *K. pneumoniae* peritonitis followed by IP injection of iNEUs or PBS (*n* = 8). Right, changes in temperature and body weight before and 12 h after injection of cells and *K. pneumoniae*. ***P < 0.001. **C** Kaplan–Meier survival curve of neutrophil-depleted BALB/c mice injected with *E. coli* (*n* = 8, left) or *P. aeruginosa* (*n* = 7, right) with or without iNEUs by IP injection

We then examined whether adoptive transfusion of iNEUs could protect the severe infection in mice. We first examined the survival of iNEUs transfused in either normal or immune-deficient mice. The transfused iNEUs could be detected in both the normal BALB/c and immune-deficient mice in 24 h after transfusion (Fig. S1D, E). To largely mimic the neutropenia, the BALB/c mice were firstly treated with anti-Ly6G antibody by intraperitoneal (IP) injection to impair their normal neutrophil functions (Asselin-Paturel et al. 2001) (Fig. S1A-C) and then injected the bacteria with or without iNEUs by IP in an E:T ratio of 1:10 (Fig. 5A). The body temperatures, body weight as well as the survival rate were measured following the injection. *K. pneumoniae* infections could cause various health problems such as liver abscesses, bacteremia, pneumonia, particularly in immunocompromised patients (Liu et al. 2024; Miller and Arias 2024). We IP-injected *K. pneumoniae* with or without iNEUs in BALB/c mice that were pre-treated with anti-Ly6G antibody and observed a strong benefit for the mice by iNEUs transfusion (Fig. 5B). Strikingly, infected with the same amount of bacteria, all control group mice injected with only the bacteria died within 24 h while no mice in the iNEUs transfusion group died in the following 14 days (Fig. 5B). Accordingly, the body temperature was rapidly reduced in mice upon bacterial infection but rescued by iNEUs transfusion (Fig. 5B). Whole body weight loss showed slight difference between two groups (Fig. 5B). These data indicate that iNEUs showed substantial antibacterial function in vivo to protect lethal infections. In addition to *K. pneumoniae, P. aeruginosa* and *E. coli* are also common bacteria to cause severe health problems in immunocompromised patients. Similarly, iNEUs transfusion in mice infected with these two bacteria showed improved overall survival (OS) (Fig. 5C) albeit that the same number of different bacteria caused differential lethal rate in BALB/c mice.

**Fig. 6:**
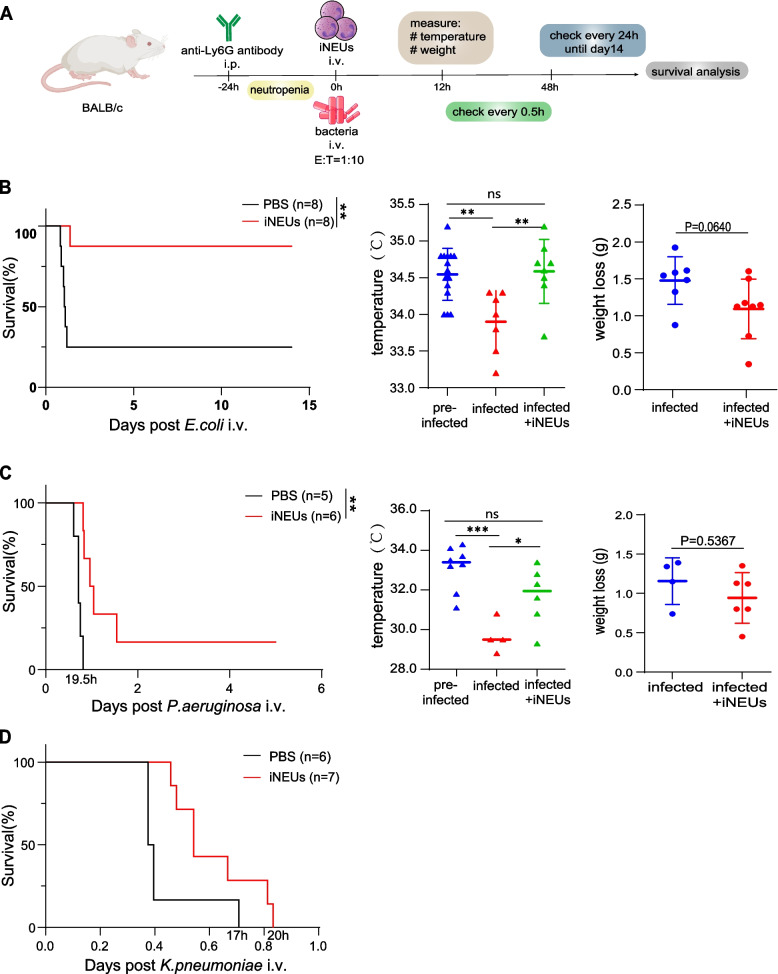
iNEUs promote the survival of lethal bacteremia mice. **A** Schematic of the experiment design. The mice were treated by anti-Ly6G antibody to impair neutrophils and were intravenously injected with bacteria with or without iNEUs. Dose: 3 × 10^8^ CFUs *E. coli*, 2 × 10^8^ CFUs *P. aeruginosa,* or 2 × 10.^9^ CFUs *K. pneumoniae*. The number of cells was one tenth of bacteria, E: T = 1:10. **B** iNEUs transfusion promotes survival of mice with lethal *E. coli* bacteremia. Left, Kaplan–Meier survival curve of neutrophil-depleted BALB/c mice with *E. coli* followed by IV injection of iNEUs or PBS (*n* = 8). Right, changes in temperature and body weight before and 19 h after injection of cells and *E. coli*. **P < 0.01, ns, not significant. **C** iNEUs transfusion promotes survival of mice with lethal *P. aeruginosa* bacteremia. Left, Kaplan–Meier survival curve of neutrophil-depleted BALB/c mice with *P. aeruginosa* followed by IV injection of iNEUs (*n* = 6) or PBS (*n* = 5). Right, changes in temperature and body weight before and 15 h after injection of cells and *P. aeruginosa*. *P < 0.05, **P < 0.01, ***P < 0.001, ns, not significant. **D** iNEUs transfusion promotes survival of mice with lethal *K. pneumoniae* bacteremia. Kaplan–Meier survival curve of neutrophil-depleted BALB/c mice with *K. pneumoniae* followed by IV injection of iNEUs (*n* = 7) or PBS (*n* = 6)

Bacteremia refers to bacterial infection in bloodstream usually causes severe or lethal problems in neutropenia patients such as systematic inflammatory syndrome, septic shock, multiple organ dysfunction syndrome, etc. We examined whether transfusion of iNEUs could protect the systematic blood infection in neutropenia mice (Fig. 6A). Similarly, the BALB/c mice were first treated with anti-Ly6G antibody by IP to induce neutropenia and then performed intravenous (IV) injection of different bacteria with or without iNEUs in an E: T ratio of 1:10 (Fig. 6A). Strikingly, iNEUs transfusion in neutropenia mice showed improved OS with infection of different common bacteria such as *P. aeruginosa* and *E. coli* as well as *K.pneumoniae* (Fig. 6B-D). iNEUs transfusion also prevented body weight and temperature loss in infected mice (Fig. 6B-D). Notably, iNEUs showed the best protection on the mice with lethal infection of E. coli (Fig. 6B) that only 1 mouse died in iNEUs group versus 7 mice died in non-iNEUs group (Fig. 6B). The infected mice with iNEUs transfusion also were much more active compared with non-iNEUs group (Supplemental Movie 1). Together, these data demonstrate that the adoptive transfusion of iNEUs is beneficial and promotes the survival in mice with neutrophil dysfunction upon lethel infection.

## Discussion

We provide the data to show that neutrophils from human iPSCs hold typical antibacterial potency against different bacteria including gram positive and negative ones both in vitro and in vivo. Neutrophils are important immune cells that are not only have a well-known antimicrobial potency but also play critical roles to modulate innate and adaptive immune response (Kolaczkowska and Kubes 2013; Moffat and Gwyer Findlay 2024; Wilson et al., 2022). Dysfunction of neutrophils usually leads to severe health problems not only infection but immune and inflammatory diseases. Neutropenia is very common in many patients with stem cell transplantation, chemotherapy, leukemia, etc. and a major risk factor for severe infections. Neutrophil transfusions have been attempted to treat neutropenia patients, but the efficacy in patients for neutrophil transfusion remains less clear. However, the data from animal models showed positive outcome for neutrophil transfusion (Miyauchi et al. 2021; Trump et al. 2019). The critical factor to obtain a positive outcome in neutrophil transfusion might be using high doses of functional neutrophils. Thus, generation of neutrophils from hiPSCs is highly promising and has been attempted by different groups (Asselin-Paturel et al. 2001; Brok-Volchanskaya et al. 2019; Casanova et al. 2024; Chang et al. 2022; Estcourt et al. 2015; Gu et al. 2024; Hopff et al. 2024; Kang et al. 2024). However, whether the hiPSCs derived iNEUs have anti-microbial potencies against different bacteria has not been extensively analyzed. Here, based on a previously reported blood differentiation protocol, we develop a defined and scalable approach to generate iNEUs. The generated iNEUs exhibit typical neutrophil characters related to antibacterial capability, such as NETs formation, ROS production and phagocytosis. Importantly, iNEUs display a strong killing potency against various gram positive or negative bacteria such as *K. pneumoniae, P. aeruginosa, E. coli* and *S. aureus*. Strikingly, transfusions of iNEUs in mice with impaired neutrophil function largely promote their survival in lethal infection of different bacteria.

Bacteria such as *K. pneumoniae, P. aeruginosa, E. coli* and *S. aureus* are very common bacteria to cause severe infections particularly in hospitalized patients who are immunocompromised and anti-biotic resistant, such as cancer patients under chemotherapy. hiPSCs derived iNEUs provide an off-the-shelf way to produce functional neutrophils to treat these patients. Another advantage for hiPSCs derived iNEUs is that the gene editing could be easy to perform in hiPSCs to promote iNEUs function. It has been known that neutrophils kill gram positive and negative bacteria through different mechanisms. Our data showed that iNEUs did exhibit differential capabilities against different bacteria both in vivo and in vitro (Figs. 3, 4, 5). Thus, it would be worth to extensively investigate the bacteria killing mechanism in neutrophils in order to generate functionally enhanced iNEUs or identify drugs to directly target and promote neutrophil functions, for which purposes, differentiation of iNEUs from iPSCs would be highly valuable.

## Methods

### Cell lines and culture

The hiPSCs (UH10) cell line, derived from the umbilical cord blood of a healthy Chinese female, was identified through short tandem repeat (STR) analysis and confirmed to be free of mycoplasma, bacteria, and fungi during cell culture and differentiation. The hiPSCs were maintained in mTeSR1 medium (85850; Stemcell Technologies) supplemented with 1% penicillin–streptomycin (SV30010; Hyclone) on Matrigel-coated plates (1:200 dilution; 354234; Corning) at 37°C in 5% CO_2_ and passaged with 0.5 mM ethylene Diamine Tetra acetic Acid (EDTA) every 3~4 days.

### Hematopoietic differentiation

The hiPSCs were dissociated by Accutase (A6964-500 mL; Sigma) and plated onto growth factor reduced Matrigel-coated plates with 0.1 μM thiazovivin (S1459-5 mg; Selleck). Various cytokines and inhibitors were then added as follows, with the medium changed daily: At day 0, 100 ng/mL BMP4 (120-05ET; Peprotech), 100 ng/mL ActivinA (120–14–50; Sino Biological Inc.), 50 ng/mL bFGF (10,014-HNAE; Sino Biological Inc.) and 50 μM CHIR99021 (S1263-5 mg; Selleck). On day 1, 100 ng/mL BMP, 5 μM A8301 (HY-10432; Selleck), and 5 μM IWR-1-endo (S7086-10 mg; Selleck). On days 2~4, 100 ng/mL VEGF (10008-HNAB-100; Sino Biological Inc.) and 100 ng/mL bFGF. On day 4 and beyond,, 100 ng/mL bFGF, 50 μM SB431542 (S1067-50 mg; Selleck), 10 ng/mL TPO(13194-H08B-100; Sino Biological Inc.), 50 ng/mL Flt3L (300–19–100; PeproTech), and 10 ng/mLl IL6 (10395-HNAE-100; Sino Biological Inc.). Basal medium (BM): DMEM/F12 (GIBCO) + 1% penicillin–streptomycin (Hyclone) + 10 μg/mL vitamin C (Vc, 2-Phospho-L-ascorbic acid trisodium salt solution, Sigma). Floating HSPCs were collected for neutrophil differentiation from day 10.

### Neutrophil differentiation

On days 0~5, hematopoietic stem and progenitor cells (HSPCs) were massively amplified in StemPro™−34 SFM medium (10639011; life technologies) supplemented with 1% penicillin–streptomycin, 10% FBS (SE100-B; VISTECH), 1% GlutaMAX (35050061; Gibco), 50 ng/mL G-CSF (10007-HNCH-5; Sino Biological), and 20 ng/mL hIL3. On days 5~9, the cells were cultured in RPMI 1640 medium (C11875500BT; ThermoFisher) supplemented with 1% penicillin–streptomycin, 10% FBS, 1% GlutaMAX, and 100 ng/mL G-CSF. The medium was changed every 2~–3 days. Cells differentiated up to day 9 were collected for subsequent experimental assays.

### Flow cytometry

The cells were suspended in DPBS (C14190500BT; ThermoFisher) with 2% FBS and incubated with multicolor antibody combinations for 20 min at 4°C. After incubation, the marker expressions were analyzed by the CytoFLEX Flow Cytometer (CytoFlox-S; Beckman). The following antibodies were used to analyze cell surface markers: APC-conjugated anti-human CD11b (301310; Biolegend), APC-conjugated anti-human APC-conjugated anti-human CD15 (561716; BD Pharmingen), PE-conjugated anti-human CD16 (555407; BD Biosciences), PE-conjugated anti-human CD62L (E-AB-F1051D, Elabscience), FITC-conjugated anti-human CD18 (101405; Biolegend), Percpcy5.5-conjugated anti-human CD66b (396914; BD Pharmingen).

### Wright-Giemsa stain

A total of 6 × 10^5^ Cells were harvested and resuspended in 150 mL DPBS for Cytospin centrifuge (500 g, 3 min, TXD3; cence). The cells were then stained with Wright-Giemsa Stain solution (BA-4010; Baso) following the manufacturer’s instructions. Cell morphology was subsequently observed under a light microscope (IX73, Olympus).

### Phagocytosis assay

Phagocytosis was assessed using Carboxylate-modified red fluorescent latex beads with a mean diameter of 1 mm (L3030; Sigma-Aldrich). In a 24-well plate, 1 × 10^5^ cells were cultured in 400 μL RPMI-1640 medium supplemented with 10% FBS and 1% penicillin–streptomycin. The latex beads were added at a dilution of 200:1 and incubated at 37℃ for 2 h. After incubation, the cells were washed twice with PBS containing 2% FBS and then analyzed by flow cytometry.

### Reactive oxygen species (ROS) production assay

A total of 5 × 10^5^ cells were seeded into 24-well plate and stimulated with 100 nM PMA for 30 min. After stimulation, H2DCFDA (KGAF018; KeygenBiotech) was added at a 1:500 concentration. The cells were then resuspended by centrifugation in serum-free medium and incubated with H_2_DCFDA for 30 min at 37℃. Following repeated washing in DPBS, the cells were analyzed by flow cytometry.

### Chemotaxis assay

PBS, either alone or containing 10% FBS, was added to the lower chamber of 24-well transwell plates (8 μm pore size, 6.5 mm diameter, 3422; Corning). A total of 2 × 10^5^ cells were seeded onto the upper chamber with 200μL of FBS-free medium. After 24 h of incubation, the upper chamber was removed, and the cells in the lower chamber were collected and mixed. The cells were then photographed under a light microscope (IX73, Olympus), and the number of cells in the field of view was counted.

### NETosis assay

A 500 µl solution of 0.25 mg/mLl poly-L-lysine (P856789; Macklin) was added to a 24-well plate containing a confocal crawler (801,010; NEST) and allowed to stand for 1 h. After aspirating the poly-L-lysine, 3 × 10^5^ cells were added and stimulated with 100 nM PMA for 7 h. The cells were then incubated with 4% paraformaldehyde (wj0012; GenXion Biotechnology) for 20 min. Subsequently, the cells were stained overnight at 4°C with an anti-MPO antibody (HPA021147; Sigma-Aldrich) in PBS containing 10% FBS and 0.3% Triton X-100 (T8787; Sigma-Aldrich). The next day, the cells were stained with a secondary antibody, goat anti-rabbit IgG G & Rb (H + L) Alexa Fluor 568 (A-11011; ThermoFisher), for 1 h in PBS with 1% FBS at room temperature. Before mounting, the nuclei were counterstained with 4’,6-diamidino-2-phenylindole (DAPI; D9542; Sigma) for an additional 5 min. Finally, the cells were imaged using a confocal laser scanning microscope (Zeiss LSM 710).

### Real-time quantitative PCR

The total RNA was extraction by the RaPure Total RNA Micro Kit (R4012-03–250 test; Magen) following the manufacturer’s instructions. The reverse transcription was performed by the HiScript II 1st Strand cDNA Synthesis Kit (R211-02; Vazyme). Quantitative real-time PCRs were carried out in ChamQ SYBR qPCR Master Mix (Q711-02; Vazyme) with a CFX96 machine (Bio-Rad). The results were normalized to GAPDH**.** The primers are shown below:

- IL1A-PF: 5’-TGGTAGTAGCAACCAACGGGA-3’
- IL1A-PR: 5’-ACTTTGATTGAGGGCGTCATTC-3’
- IL1B-PF: 5’-ATGATGGCTTATTACAGTGGCAA-3’
- IL1B-PR: 5’-GTCGGAGATTCGTAGCTGGA-3’
- CXCL8-PF: 5’-ACTGAGAGTGATTGAGAGTGGAC-3’
- CXCL8-PR: 5’-AACCCTCTGCACCCAGTTTTC-3’
- CCL4-PF: 5’-CTGTGCTGATCCCAGTGAATC-3’
- CCL4-PR: 5’-TCAGTTCAGTTCCAGGTCATACA-3’

### Bacterial strains and culture media

The bacterial strains used in this study include selectable marker-free autoluminescent *P. aeruginosa* (SfAlPa), selectable marker-free autoluminescent *K. pneumoniae* (SfAlKp), *E. coli* strain T1 and *S. aureus* ATCC25923. The bacterial strains all were obtained from Tianyu Zhang’s lab of Guangzhou Institutes of Biomedicine and Health and grown at 37 °C in Luria–Bertani (LB) broth.

### Killing assay of autoluminescent bacteria for RLUs detection

Cells were diluted with antibiotic-free RPMI 1640 medium to a concentration of 5 × 10^5^ cells per mL. Bacteria were placed in a luminometer (Promega) to measure the RLUs and diluted to 5 × 10^5^ RLU per mL with antibiotic-free RPMI 1640 medium. In a 96-well plate, 100 μL of the bacterial suspension was added to 100 μL of the cell suspension. The RLUs were detected using a multi-label microplate detector (EnVision; PerkinElmer) at 0, 2, and 4 h of co-culture.

### Killing assay of bacteria for CFUs detection

Cells were diluted with antibiotic-free RPMI 1640 medium to a concentration of 2 × 10^4^ cells per mL. Bacteria were placed in a bio-spectrophotometer (BioPhotometer; Eppendorf) to measure optical density at 600 nm (OD600) and then diluted to 2 × 10^5^ CFUs per mL with antibiotic-free RPMI 1640 medium based on the standard curve. At 0 h, 100 μL of the bacteria suspension was added to 100 μL of cell suspension in a 96-well plate. After 2 h of co-culture, the mixture was subjected to serial dilution and plated on LB agar plates. The number of colonies was counted after overnight incubation.

### Animal experiments

The animal experiments have been approved by the Ethical Committee on Animal Experiments at Guangzhou Institutes of Biomedicine and Health, Chinese Academy of Sciences (GIBH, CAS). Animal experiments schemas are shown in detail in each relevant figure. As for the acute lethal peritonitis model**,** neutrophils were depleted with anti-Ly6G antibody (BE0075-1; Biocell). 6-week-old BALB/c mice were then intraperitoneally injected with bacteria (approximately 5 × 10^8^ CFUs *E. coli*, 5 × 10^7^ CFUs *P. aeruginosa,* or 5 × 10^7^ CFUs *K. pneumoniae* respectively) and PBS or neutrophils (at a concentration one-tenth that of the bacterial load)*.* The bacterial concentrations were determined by autofluorescence. The mice were monitored for survival, with observations made at 1-h intervals for the first 48 h and then daily. As for acute lethal bacteremia model and neutrophil therapy, neutrophils were depleted with anti-Ly6G antibody. 6-week-old BALB/c mice were then intravenously injected with bacteria (approximately 3 × 10^8^ CFUs of *E. coli*, 2 × 10^8^ CFUs of *P. aeruginosa*, or 2 × 10^9^ CFUs of *K. pneumoniae* per mouse respectively) and PBS or neutrophils (at a concentration one-tenth that of the bacterial load). The bacterial concentrations were determined by autofluorescence. The mice were monitored for survival, with observations made at 1-h intervals for the first 48 h and then daily. All mice purchased from GemPharmatech and bred in the SPF-grade animal care facility of GIBH, and procedures were approved by the GIBH Animal Care and Use Committee (No. 2023037).

### Temperature measurement in mice

The oral temperature was opted to be measured instead. The metal probe of the thermometer was inserted approximately 2 cm into the mouse's mouth. The final temperature was recorded once the thermometer reading stabilized for at least 10 s.

### Statistical analysis

All the statistics were performed in GraphPad Prism 9 (GraphPad). All the data reported as mean ± SD from at least three independent experiments. The level of significance between samples was analyzed unpaired t-test. Asterisks indicate the following significance: * indicates *P* < 0.05, ** indicates *P* < 0.01, *** indicates *P* < 0.001.

## Supplementary Information

Additional file 1: Supplemental Figure 1. Anti-Ly6G antibody depleted neutrophils in mice.

Additional file 2: Supplemental Movie 1. iNEUs promotes activation in mice with lethal bacteremia. Activity in mice with *E. coli* bacteremia followed by IV injection of iNEUs (below) or PBS (above). Shot on iPhone 12.

## Data Availability

The RNA-seq data have been deposited in the GEO Database: GSE290954. The datasets used and/or analysed during the current study are available from the corresponding author on reasonable request.

## Abbreviations

hiPSCs: Human induced pluripotent stem cells
iNEUs: HiPSCs derived neutrophils
NETs: Neutrophil extracellular traps
NeuPs: Neutrophil-primed progenitors
iHSPCs: hiPSCs derived hematopoietic stem/progenitor cells
EHT: Endothelial-hematopoietic-transition
FBS: Fetal bovine serum
G+: Gram positive
G-: Gram negative
E: T: Effector-to-target
OS: Overall survival
STR: Short tandem repeat
EDTA: Ethylene Diamine Tetra acetic Acid
DAPI: 4’,6-Diamidino-2-phenylindole
PMA: Phorbol myristate acetate
ROS: Reactive oxygen species
