# Correction: Human induced pluripotent stem cells derived neutrophils display strong anti-microbial potencies

**DOI:** 10.1186/s13619-025-00254-w

**Published:** 2025-07-10

**Authors:** Xing Hu, Baoqiang Kang, Mingquan Wang, Huaisong Lin, Zhiyong Liu, Zhishuai Zhang, Jiaming Gu, Yuchan Mai, Xinrui Guo, Wanli Ma, Han Yan, Shuoting Wang, Jingxi Huang, Junwei Wang, Jian Zhang, Tianyu Zhang, Bo Feng, Yanling Zhu, Guangjin Pan

**Affiliations:** 1 National Key Laboratory of Immune Response and Immunotherapy, Guangzhou Institutes of Biomedicine and Health, Chinese Academy of Sciences, Guangzhou, 510530 China; 2 University of Chinese Academy of Sciences, Beijing, 100049 China; 3 Centre for Regenerative Medicine and Health, Hong Kong Institute of Science and Innovation, Chinese Academy of Sciences, Hong Kong, China; 4 Guangdong Provincial Key Laboratory of Stem Cell and Regenerative Medicine, Guangdong-Hong Kong Joint Laboratory for Stem Cell and Regenerative Medicine, Center for Development and Regeneration, Guangzhou Institutes of Biomedicine and Health, Chinese Academy of Sciences, Guangzhou, 510,530 China; 5 GIBH-HKU Guangdong-Hong Kong Stem Cell and Regenerative Medicine Research Centre, GIBH-CUHK Joint Research Laboratory on Stem Cell and Regenerative Medicine, Guangzhou Institutes of Biomedicine and Health, Chinese Academy of Sciences, Guangzhou, 510,530 China; 6 School of Biomedical Sciences, Faculty of Medicine, CUHK-GIBH CAS Joint Research Laboratory on Stem Cell and Regenerative Medicine, The Chinese University of Hong Kong, Room 105 A, Lo Kwee-Seong Integrated Biomedical Sciences Building, Area 39, Shatin, NT, Hong Kong, SAR China; 7 The Third Affiliated Hospital of Sun Yat-Sen University, Guangzhou, 510,000 China

**Correction: Cell Regeneration 14, 8 (2025)**

**https://doi.org/10.1186/s13619-025-00227-z**

Following publication of the original article (Hu et al. 2025), the authors reported an error in Fig. 1E, the FACS data of surface markers CD11b and CD18 on iNEUs were mistakenly duplicated. Upon checking the original raw data, this error was caused by accidentally duplicating the same picture when formatting the figure.

The correct Fig. 1 has been provided in this Correction. These corrections do not affect the conclusions of this study.

The incorrect Fig. 1 is:

**Fig. 1:**
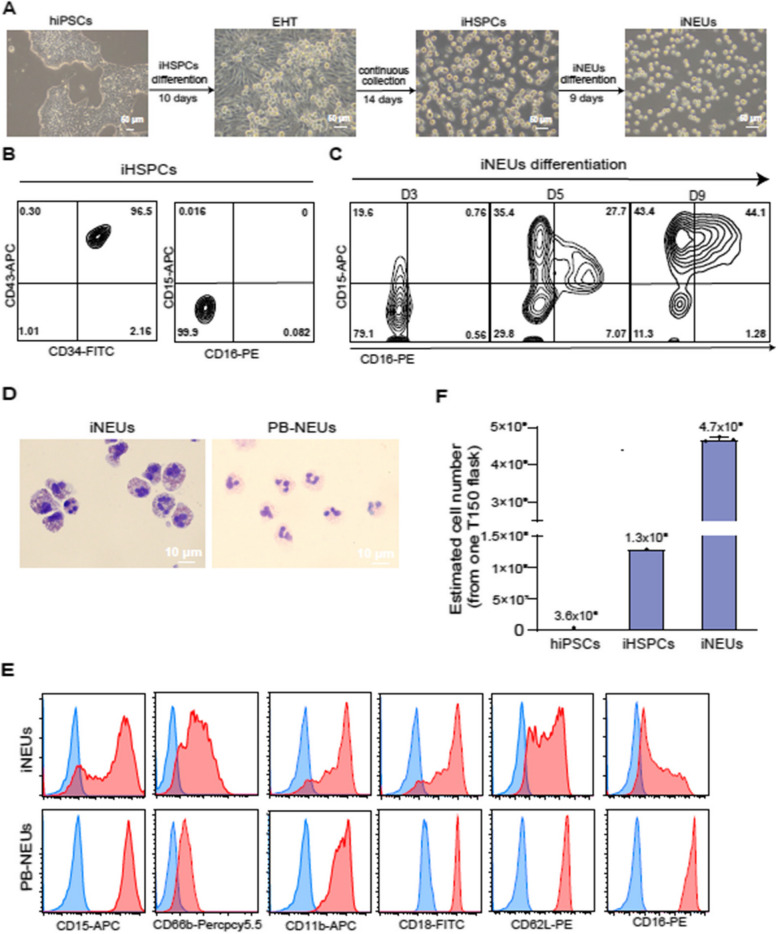
Generation of neutrophils from hiPSCs. **A** Representative brightfield images of different stages of iNEUs differentiation from hiPSCs. Scale bar, 50 μm. **B**, **C** Representative FACS data of indicated markers on iHSPCs (**B**) and iNEUs differentiation at different time points (**C**). **D** Wright–Giemsa images of iNEUs and PB-NEUs. Scale bar, 10 μm. **E** Representative FACS data of indicated surface markers (CD15, CD66b, CD11b, CD18, CD62L and CD16; red filled) on iNEUs and PB-NEUs (blue, respective isotype control). **F** Estimated number of iHSPCs and iNEUs obtained from 3 × 10^6^ hiPSCs cultured in 1 T150 flask

The correct Fig. 1 is:

**Fig. 1:**
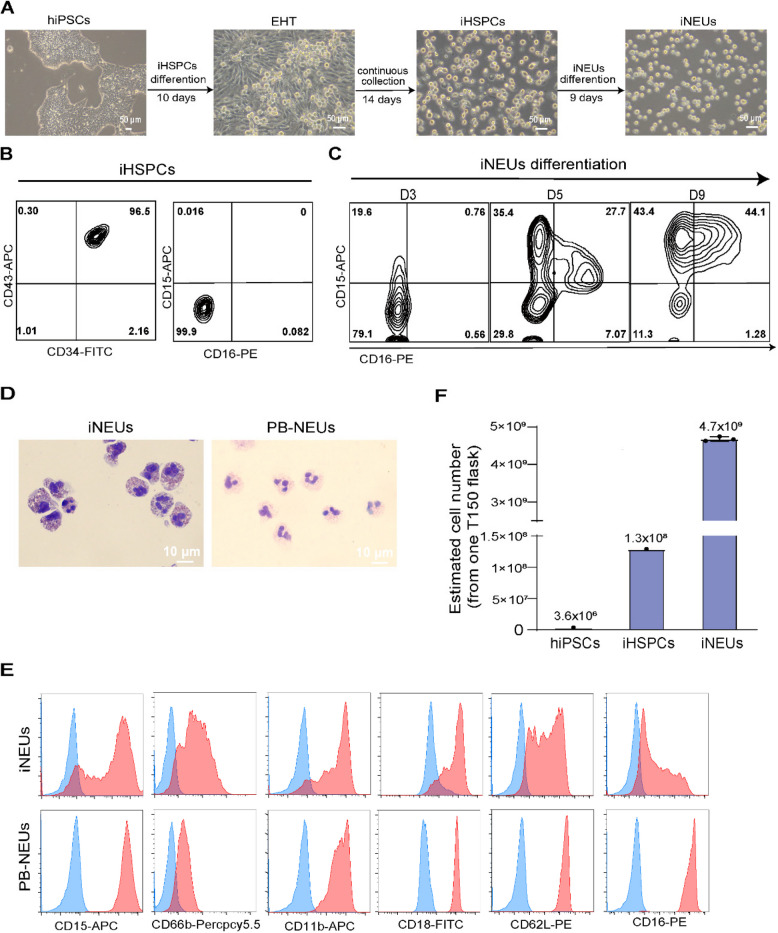
Generation of neutrophils from hiPSCs. **A** Representative brightfield images of different stages of iNEUs differentiation from hiPSCs. Scale bar, 50 μm. **B**, **C** Representative FACS data of indicated markers on iHSPCs (**B**) and iNEUs differentiation at different time points (**C**). **D** Wright–Giemsa images of iNEUs and PB-NEUs. Scale bar, 10 μm. **E** Representative FACS data of indicated surface markers (CD15, CD66b, CD11b, CD18, CD62L and CD16; red filled) on iNEUs and PB-NEUs (blue, respective isotype control). **F** Estimated number of iHSPCs and iNEUs obtained from 3 × 10^6^ hiPSCs cultured in 1 T150 flask

The original article (Hu et al. 2025) has been corrected.
