# Supplementary figures and images for: Human induced pluripotent stem cells derived neutrophils display strong anti-microbial potencies
Source: Cell Regen. 2025 Mar 21;14:8. doi: 10.1186/s13619-025-00227-z (PMC11926315; doi:10.1186/s13619-025-00227-z)

Supplemental Figure.S1

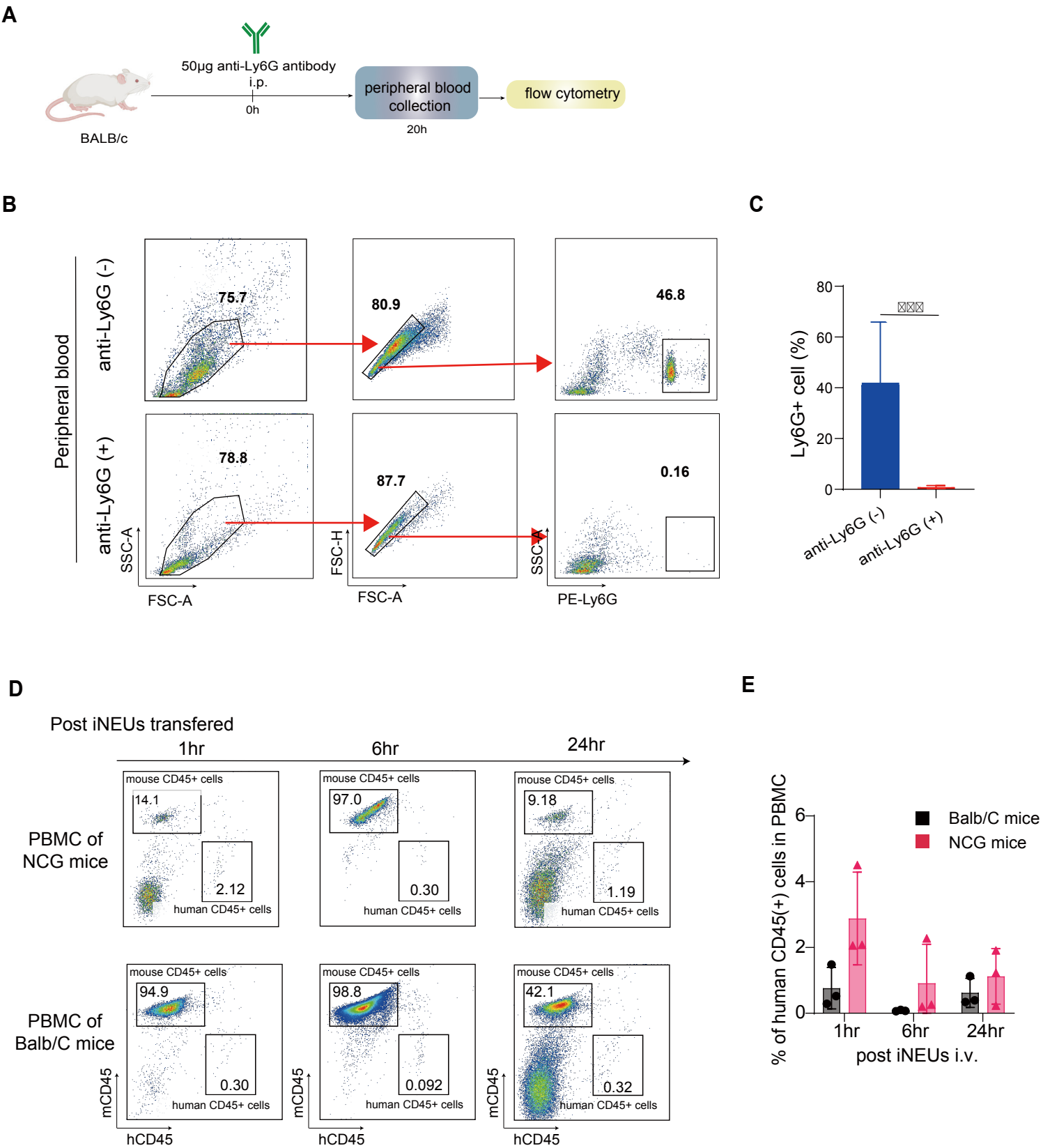

Supplement: Supplementary file 1 — Additional file 1: Supplemental Figure 1. Anti-Ly6G antibody depleted neutrophils in mice. [file 13619_2025_227_MOESM1_ESM.pdf]
